# Impregnating hessian strips with the volatile pyrethroid transfluthrin prevents outdoor exposure to vectors of malaria and lymphatic filariasis in urban Dar es Salaam, Tanzania

**DOI:** 10.1186/s13071-015-0937-8

**Published:** 2015-06-12

**Authors:** Nicodem J. Govella, Sheila B. Ogoma, John Paliga, Prosper P. Chaki, Gerry Killeen

**Affiliations:** Ifakara Health Institute, Environmental Health and Ecological Sciences Thematic Group, Coordination Office, P.O Box 78373, Kiko Avenue, Mikocheni, Dar es Salaam United Republic of Tanzania; US Army Medical Research Unit Kenya-Walter Reed Project, P.O. Box 54, Kisumu, Kenya; Department of Vector Biology, Liverpool School of Tropical Medicine, Pembroke Place, Liverpool, L3 5QA United Kingdom

**Keywords:** Filariasis, Malaria, Mosquitoes, Outdoor transmission, Repellent, Transfluthrin, Hessian strip, Vector control, Diversion

## Abstract

**Background:**

Semi-field trials using laboratory-reared *Anopheles arabiensis* have shown that, delivering the volatile pyrethroid transfluthrin by absorption into hessian strips, consistently provided > 99 % human protective efficacy against bites for 6 months without retreating. Here the impact of this approach upon human exposure to wild populations of vectors for both malaria and filariasis under full field conditions is assessed for the first time.

**Methods:**

Transfluthrin-treated and untreated strips were placed around human volunteers conducting human landing catch in an outdoor environment in urban Dar es Salaam, where much human exposure to malaria and filariasis transmission occurs outdoors. The experiment was replicated 9 times at 16 outdoor catching stations in 4 distinct locations over 72 working nights between May and August 2012.

**Results:**

Overall, the treated hessian strips conferred 99 % protection against *An. gambiae* (1 bite versus 159) and 92 % protection against *Culex spp.* (1478 bites versus 18,602). No decline in efficacy over the course of the study could be detected for the very sparse populations of *An. gambiae* (*P* = 0.32) and only a slow efficacy decline was observed for *Culex spp.* (*P* < 0.001), with protection remaining satisfactory over 3 months after strip treatment. Diversion of mosquitoes to unprotected humans in nearby houses was neither detected for *An. gambiae* (*P* = 0.152) nor for *Culex spp.* (Relative rate, [95 % CI] = 1.03, [0.95, 1.11], *P* = 0.499).

**Conclusion:**

While this study raises more questions than it answers, the presented evidence of high protection over long periods suggest this technology may have potential for preventing outdoor transmission of malaria, lymphatic filariasis and other vector-borne pathogens.

## Background

Malaria remains the most important parasitic disease globally, and in Africa particularly where the principal vectors are species of the *Anopheles gambiae* complex and *An. funestus* group [1, 2]. These mosquito species are distributed all across tropical Africa where they also act as vectors of the *Wuchereria bancrofti* parasites that cause Lymphatic Filariasis (LF). *Culex spp.* are also responsible for LF transmission, especially the abundant populations of *Culex quinquefasciatus* that often proliferate in sanitation systems of human settlements [3–6]. While recent effort to eliminate transmission of both malaria and LF, by combining curative drugs with indoor vector control measures such as long-lasting insecticidal nets (LLINs) and indoor residual spraying (IRS), have successfully reduced the infection burden of these two diseases [5–7], elimination of either is probably impossible without additional interventions to prevent outdoor transmission in particular [4, 8–12].

Following recent scale up of LLINs and IRS [7] several populations of vectors for malaria and LF, have been observed to bite more at dusk and dawn when many residents are awake and active outdoors than would be normal for these species in settings scattered all across Africa [13–17] and beyond [18, 19]. However, with some exceptions [20, 21] it is not entirely clear whether these vector populations were truly genetically homogenous in the first place and really have been selected for heritably modified behavior traits, rather than just for altered composition of taxonomically distinct populations with different behavioural characteristics or, even more simply, by a manifestation of phenotypic plasticity of pre-existing traits [10, 22, 23]. Such altered or atypical biting times to avoid periods when humans are asleep indoors and may be protected by LLINs or IRS obviously reduce the effectiveness of these key top-priority interventions [23, 24] and highlights the importance of complementary outdoor tools to combat both malaria and LF.

Volatile insecticides are one potential supplementary vector control tool which could complement LLINs and IRS because they have a different mode of action which lends itself to outdoor application. Volatile active ingredients with repellent or toxic modes of action may be delivered in vapour phase so they protect spaces by diffusion through the air, rather than relying on mosquito contact with structural surfaces in the way as conventional, solid-phase contact insecticides [25, 26]. So while LLINs and IRS require an enclosing structure, like a house, shelter or net, volatile insecticides can be readily applied in any indoor or outdoor space [25, 26].

A variety of different methods for dispensing volatile insecticides exist [27–29], but most of these formats require too high a frequency of replacement to be practical or affordable in the low income tropical countries. Fortunately, it has recently been shown that, it is possible to disperse the volatile pyrethroid transfluthrin by absorbing it into strips of hessian [30]. Such transfluthrin-treated strips of hessian can provide very high levels (>99 %) of human protection against mosquito bites for at least 6 months after treatment under experimental conditions [30]. Furthermore, this delivery format does not require external source of energy, such as combustion or electricity to volatilize the active ingredient [30]. Hessian, or burlap as it is known in North America, is a robust fabric made from woven fine jute fibers, which is widely used across the tropics for storage and transport of bulk goods. However, while the results of the first study demonstrating the potential of this approach [30] are encouraging, these experiments were conducted using laboratory-reared mosquitoes from a single mosquito species (*Anopheles arabiensis*) under semi-field conditions in caged enclosures. Therefore, these reported estimates of protective efficacy may not be fully representative of real life situations, where wind and other environmental conditions may affect performance of these prototype devices for vapour phase insecticide delivery against taxonomically and behaviourally diverse populations of free-flying wild mosquito. Also, while transfluthrin has been widely described and marketed as a spatial repellent [25] indicate that it functions by reducing or delaying blood feeding of exposed mosquitoes without hindering attraction to humans . It is therefore unclear whether this active ingredient will cause inequitable diversion of mosquitoes that survive transfluthrin exposure from protected to unprotected individuals, in the same way that topical repellents do.

This study was therefore conducted to quantify the protective effect of these transfluthrin-impregnated hessian strips against outdoor exposure to both malaria and LF vectors, as well as diversion to unprotected non-users in nearby houses, under full field conditions in the Tanzanian city of Dar es Salaam, where substantial outdoor exposure is known to occur [16, 17, 31] and physiological resistance to pyrethroids appears to be emerging [32, 33]. Dar es Salaam is a particularly interesting study setting because stable malaria transmission is still mediated by sparse vector populations that have been suppressed by high coverage of window screening, insecticide-treated nets and, in some areas, larviciding [17, 34–36], but nevertheless persists, presumably to a large extent because they tend to feed outdoors and in the early evenings [16, 17, 31] more frequently than is typical of African vectors [11, 37]. Furthermore, dense human populations with poor sanitation infrastructure result in very dense populations of *Culex quinquefasciatus* [17, 38–40], that not only transmit LF but also cause sufficient biting nuisance to drive seasonal fluctuations in the sale of repellent mosquito coils [41], as well as high rates of housing modification to prevent mosquito entry [35].

## Methods

### Study sites

The study was conducted in four separate locations in urban Dar es Salaam, specifically in the four wards of Magomeni, Jangwani, Keko and Kurasini [42, 43], where foci with relatively high densities of mosquitoes from the *An. gambiae sensu lato* complex occur, so it is possible to catch sufficient numbers of malaria vectors to characterize their behavior [17], and experimentally evaluate the efficacy of mosquito traps [39, 44], or in this case, volatile insecticides for protecting human subjects. Members of the *An. gambiae* complex (*An. gambiae sensu stricto*, *An. arabiensis, An. merus*) and *An funestus* group (Almost exclusively *An. funestus sensu stricto*) are the principal malaria vectors in this setting, with *An. gambiae* and *An. arabiensis* being the most important [17, 40]. Recent observations indicate that *An. arabiensis* in Dar es Salaam now bite most actively at dusk so that, even amongst residents lacking LLINs or mosquito-proofed housing, at least half of human exposure to this mosquito occurs outdoors [16, 17, 31]. While the biting peak of *An. gambiae s.s.* in this setting remained consistent with that of classical reports [37, 45], it prefers to feed outdoor so this is where more than a quarter of exposure to this most locally important malaria vector occurs, even among residents lacking LLINs or mosquito-proofed houses [16, 17, 31]. Note that what determines where most transmission occurs is the distribution of behavioural interactions between humans and mosquitoes over time and space across the entire night, rather than just the biting behavior preferences of the vector alone [16, 37, 46]. Additionally note that, in addition to these behavioural resistance or resilience [22, 23] traits, physiological resistance to pyrethroids was also apparently emerging among local populations of *An. gambiae sensu lato* in this setting at the time of this study [32, 47].

However, these *Anopheles* vectors of both malaria and LF are remarkably sparse in this setting overall, accounting for only a tiny proportion (<1 %) of the overall human biting burden and LF transmission potential in Dar es Salaam [17, 27, 31, 36, 39–41]. High densities of *Cx. quinquefasciatus* [38] cause hundreds of bites per person per night in many parts of the city [27, 31, 38–41]. Even among the small minority of residents lacking bed nets or mosquito-proofed housing, almost half of all human exposure to this highly abundant LF vector occurs outdoors [31]. Furthermore, most residents are now protected to some degree by prevention measures [35, 36], so most exposure to LF transmission and biting nuisance occurs outdoors across the population as a whole.

### Transfluthrin-treated and untreated hessian strips

**Fig. 1:**
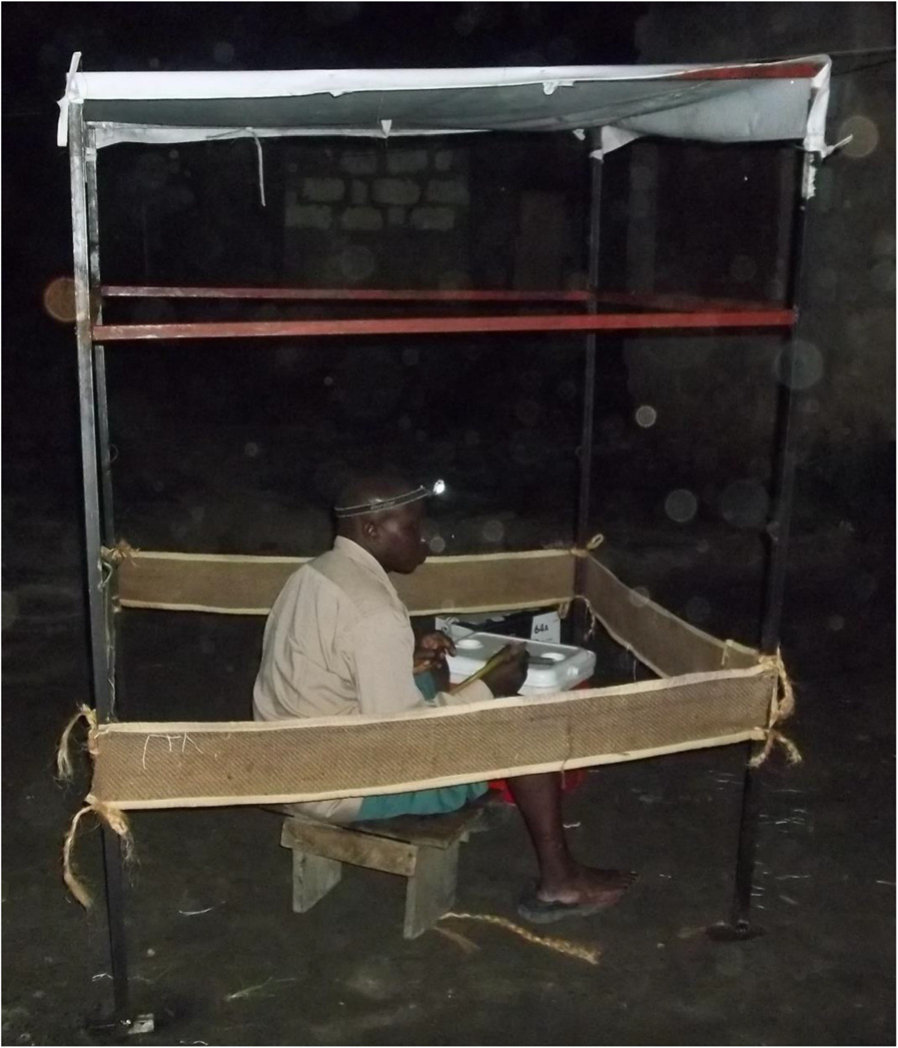
Example of a transfluthrin-treated hessian strip in use as evaluated in this study. The strip is made of fine jute fibers woven together to form sacking fabric, cut into 4 m × 0.3 m dimensions. The strip is hung 0.5 m above the ground in a square shape on four metal poles, creating a 1 m^2^ area around a human volunteer collecting mosquitoes by the human landing catch method

Hessian sacking fabric was washed and dried, and then cut into strips 4 m long and 0.3 m wide. It was then soaked with a mixture of 10 ml of transfluthrin active ingredient (97 % pure, Shenzhen Sunrising Industry Company, China) with 90 ml of Axion® liquid detergent (Orbit Chemical Industries Ltd, Nairobi and Colgate-Palmolive East Africa Ltd) diluted in 400 ml of water as previously described [30]. Transfluthrin is insoluble in water so mixing it with a detergent helped to emulsify it in a water-based bulk carrier for application to the Hessian strips. Application in this suspended form ensured uniform coverage of the full surface area of the strip with the active ingredient. Water is far more practical and safe than mineral oil or any organic solvent, and it is readily available anywhere, so it is the obvious solvent of choice for preparing affordable vector control tools that can be conveniently and affordably formulated anywhere in low income countries. The strips were then hung indoors to dry at room temperature for 2 weeks before the experiment, and this drying period was included when calculating the ages of the strips for the statistical analyses and figures. Each treated strip was suspended approximately 0.5 m above the ground on four metal poles, creating a 1 m^2^ square frame around a sitting space for a mosquito collector conducting the human landing catch (HLC) method to capture mosquitoes attempting to bite him (Fig. 1). Untreated strips were soaked with a mixture containing the diluent mixture of 400 ml of water and 90 ml of detergent but no transfluthrin. Both treated and untreated hessian strips units were each placed in outdoor catching stations approximately 5 m outside of an assigned house, with a single research participant collecting mosquitoes outdoors by HLC within the protective perimeter of the suspended strip, while another performed indoor HLC without any strip within the house.

### Human landing catch

Mosquitoes were collected with the human landing catch (HLC) method [48, 49]. This was performed by a single adult male collector at each sampling station for 45 min of each hour, allowing 15 min break for rest, refreshment and exchange of collectors between matched indoor and outdoor stations. Collections were conducted both indoors and outdoors from 7 pm to 6 am so that these data could also be used to assess mosquito behaviour patterns. Note that this study was based within the large project area of the African Vector Control New Tool (Avecnet), which primarily focused on characterizing behavior and efficacy testing of traps for sampling malaria vectors across the gradient of urbanization in Dar es Salaam.

### Experimental design

In each of four different wards (Magomeni, Jangwani, Keko and Kurasini), one block of four houses without completely screened windows, sealed eave gaps or closed ceilings, distributed approximately 50 m apart was selected. One outdoor station for HLC, in a convenient location that did not interfere with normal activities of the household, was identified and assigned approximately 5 m outside of each selected house where corresponding indoor HLC catches were to be conducted. Note that there were only two outdoor stations assigned with treated strips on each experimental night, while the remaining two were assigned untreated strips. However, neither treated nor untreated strips were used indoors so the numbers of mosquitoes caught during corresponding indoor HLCs could only have been influenced by treated strips being used by participants conducting HLC at the outdoor station immediately outside at the same time. These pairs of treated and untreated strips were swapped between the catching stations after each experimental night, using a crossover experimental design. Two nights of experimentation were therefore needed to complete one round of collection in a single block comprising four pairs of outdoor catching stations and matched indoor stations, following which the experiment moved to the next block for implementation of exactly the same crossover design. This two-night unit of experimental replication was sequentially moved through all four blocks, over a total of eight nights of sampling, so that both crossover arrangements of the treated and untreated strips were conducted at all 16 outdoors catching stations and inside all 16 houses. The collection was conducted over 72 nights of experimentation, comprising of 9 full rounds of replication across all four blocks, distributed over a period of 100 days that included 28 days off without experimentation, from the 21^st^ of May to the 31^st^ of August 2012. There were therefore a total of 144 person nights of outdoor mosquito capture, and the same number indoor, conducted over the course of the experiment, inclusive of both the treated and untreated hessian groups. Additionally note that, in matched indoor-outdoor combinations of sampling stations, collectors were exchanged between the indoor and outdoor stations after every hour, to control for the effect of variation in attractiveness and performance of collectors. Collectors were, however, not exchanged between pairs of indoor and matching outdoor catching stations within a block over any round, in order to combine the differential effects of individual attractiveness and performance with variations associated with particular house locations into a single quantifiable source of variation that could be accounted for in the statistical analysis. When strips were not used between nights of experimentation, they were stored in open, unsealed basins so that transfluthrin vapour could dissipate as it would under normal conditions and the amount of active ingredient remaining in the strips could decay at a normal rate.

### Processing of samples

Samples from all catches were sorted, counted and identified morphologically in the laboratory as either *Anopheles gambiae s.l.* or *Culex spp.* with the aid of stereo-microscope. All *An. gambiae s.l.* were stored in tubes with desiccated silica gel for subsequent polymerase chain reaction (PCR) assay [50] to determine sibling species identity.

### Data analysis

First, descriptive summaries and graphical analyses were conducted to examine daily trends in mosquito catches between transfluthrin treated and untreated strips. Then Generalized Linear Mixed Effects Models (GLMM) [51] were fitted to the data to, not only allow estimation of the protective effect of the transfluthrin vapour, but also account for substantive variance associated with multi-level confounding variables, specifically participants, catching stations and block locations, by treating them as random effects to maximize statistical power. The analyses were implemented using *R* statistical software (R x64 2.15.2) augmented with the *lme4* package.

To evaluate the effect of transfluthrin-treated hessian strips upon mosquito catches outdoor (dependent variable), the treatment status of the strips (transfluthrin-treated versus untreated) was included as a categorical independent fixed variable, while outdoors catching station (identity of associated house) nested within geographical location (blocks of houses and associated outdoor catching stations), as well as night of sampling and participant, were treated as random effects that require only a single degree of freedom each. Mosquito catch numbers are count observations, and were clearly not normally distributed, so the GLMMs applied to these data fitted this dependent variable to a Poisson distribution. In this particular analysis, only mosquito samples collected from outdoor but not indoor catching stations were used.

Essentially the same GLMM was fitted to test for the decline of protective efficacy over time, except that an interaction term between the treatment status of the strips and the number of days since the strips were treated with transfluthrin (Equivalent to days since treatment if treated but zero if not treated), was also included and treated as continuous explanatory covariate. Where this time-treatment interaction term was not significant, the model was reduced parsimoniously to the simple one described above with only treatment status included as a simple categorical fixed effect.

Similarly, to test for the possible effect of diverting attacking mosquitoes away from an outdoor user of a treated strip, so that humans inside the nearby house are bitten more, the GLMM was changed only in that the indoor mosquito catches recorded by HLC in that house on the same night was treated as the dependent variable, rather than those caught outdoors by the strip user themselves. Otherwise, the fitted GLMM was identical with the treatment status of the strip (transfluthrin treated versus untreated) used immediately outside the house being treated as a categorical independent variable, while indoor catching station (house) nested within geographical location (blocks of houses), as well as night of sampling and participant, were treated as random effects.

### Ethical approval

Research clearance was obtained from the Institutional Review Board of the Ifakara Health Institute (IHI/IRB/No. A50) and the Medical Research Coordination Committee of the National Institute of Medical Research (NIMR/HQ/R.8c/Vol.ii/125) in Tanzania. All participants were provided with drug prophylaxis (Malarone®) against malaria [52] and screened weekly for malaria parasites by rapid diagnostic test (mRDT (MAL-Pf®, ICT Diagnostics, Cape Town, South Africa, which detects histidine-rich protein II). Fortunately, no participant was found to be infected with malaria during the study but, according to the protocol, any that were would have been offered free-of-charge treatment with artemisinin-lumefantrine, (Co-Artem®, Norvatis, Basel, Switzerland), the recommended first-line treatment for malaria in Tanzania.

## Results

Respectively, a total of 370 and 47,653 *Anopheles gambiae s.l.* and *Culex spp.* were collected throughout the experiment, of which 211 and 159 *Anopheles gambiae s.l.*, as well as 31,493 and 18,602, *Culex spp.* were collected indoors and outdoors respectively. The vast majority (95 %; *n* = 158) of the 166 successful PCR-amplified specimens from the of *An. gambiae s.l.* were identified as *An. gambiae s.s.* and the remainder (5 %, *n* = 8) as *An. arabiensis*. The results presented here with respect to *An. gambiae s.l.*, therefore, effectively reflect the response of *An. gambiae s.s.* to these treatments and are represented as such.

**Fig. 2:**
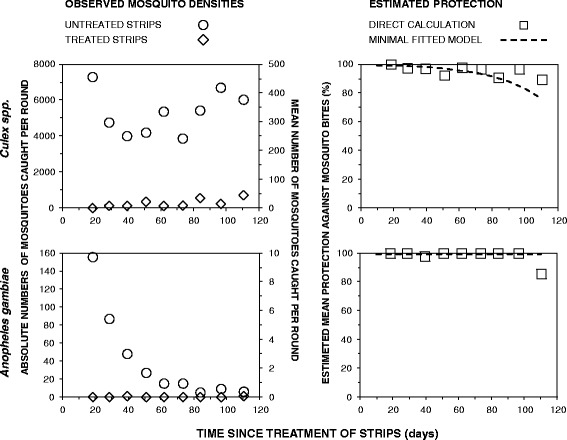
Absolute total numbers of mosquitoes caught and mean numbers caught per person night of catching for each experimental round of replication, as well as derived estimates for the protective efficacy of transfluthrin-treated hessian strips against bites. Left hand panels compare the absolute total and mean numbers of mosquitoes caught by human landing catch in both the treated and control group. Each data point represents one complete round of eight night of sampling with 16 person nights of capture for each of the control and treatment groups. Right hand panels present the calculated protective efficacy, which is defined in term of proportional reduction (*ρ*) in the number of mosquitoes caught in the test group with transfluthrin-treated strips (*N*_*t*_) relative to the negative control group with untreated negative control strips (*N*_*c*_), obtained by dividing the difference between the numbers of mosquitoes caught with treated and untreated strips by the number caught with the control strips (*ρ* = (*N*_*c*_–*N*_*t*_)/ *N*_*c*_). The trend lines represent the most parsimonious fitted generalized linear mixed models, which includes only strip treatment as a fixed effect in the case of *Anopheles gambiae* but also Poisson-linear decay of treatment effect over time in the case of *Culex spp*

Overall, the treated hessian strips conferred almost complete protection (*ρ*) against bites from *An. gambiae* (Fig. 2) as calculated on the basis of either crude numbers caught in the treatments (*N*_*t*_) and controls (*N*_*c*_) (*ρ* = (*N*_*c*_–*N*_*t*_)/*N*_*c*_ = 99.4 %; 1 caught on users of treated strips versus 159 caught by users of untreated control strips) or by GLMM analysis (*ρ* = 1-Relative Rate (RR) [95 % confidence interval (CI)] = 99.2 [93.89, 99.9 %], z = −4.924, *P* < 0.001). This level of protection against *An. gambiae* appeared to be consistent and did not detectably decay over the 100 days of the study (z = 1.11, *p* = 0.32), during which 72 nights of experimentation were conducted (Fig. 2).

In case of *Culex spp.,* the observed overall level of protection against outdoor bites was also very satisfactory (Fig. 2), regardless of whether efficacy was calculated crudely based on the absolute numbers caught (92 %; 1478 caught on users of treated strips versus 18,602 caught by users of untreated control strips) or estimated by GLMM analysis (*ρ* [95%CI] = 91.4 % [91.0, 92.0 %], z = −83.5, *P* < 0.000001). The levels of protection against *Culex spp.* declined only slightly over time (z = 26.6, *P* < 0.000001) but it remained consistently above 85 % over the three months of the study (Fig. 2).

Whereas no diversion effect from transfluthrin treated strips to a corresponding house was detected against *An. gambiae* (RR [95 % CI] = 0.31 [0.05, 2.01], z = −1.231, *P* = 0.218), this may well be due to lack of sufficient statistical power because of low mosquito densities, particularly towards the end of the study (Fig. 2). However, no evidence of diversion was apparent for the far more abundant *Culex spp.* either (RR [95 % CI] = 1.03 [0.95, 1.11], z = 0.676, *P* = 0.499).

## Discussion

The results presented here suggest that, in this particular urban African context, hessian strips impregnated with the volatile pyrethroid transfluthrin provide very high levels of personal protection against at least two important taxa of pathogen-transmitting mosquitoes. While this is only the first preliminary assessment of this prototype under full field conditions, conducted in only one setting with endemic malaria and LF, it is encouraging that the level of protection observed is approximately equivalent to that provided by insecticide-treated nets against mosquito bites occurring indoors [53–55]. Our findings are approximately consistent with those of an earlier study which evaluated this same formulation format for transfluthrin in a semi-field, large-cage system using laboratory-reared *Anopheles arabiensis* wherein ≥ 99 % reduction in mosquito bites upon humans was reported [30]. This first evaluation under full field conditions therefore confirms that hessian strips impregnated with volatile insecticides like transfluthrin may be a promising tool to combine with the current priority malaria control tools, which are threatened by behavioural resilience/resistance in the form of outdoor-biting behaviours [13, 14, 18, 19, 22], and by physiological resistance to insecticides [32, 56, 57]. It is also encouraging that, unlike better studied topical repellent formulations [58, 59], vapour phase transfluthrin does not appear to cause any detectable diversion to unprotected non-users in nearby houses.

These preliminary observations also suggest that vapor-phase pyrethroids like transfluthrin may even be useful for preventing outdoor disease transmission by populations of *An.gambiae* in which physiological resistance to this class of insecticides appears to be emerging [32, 33]. Obviously, this protective effect against pyrethroid-resistant malaria vectors will need to be thoroughly evaluated against a much wider diversity of resistant captive colonies and wild populations before vapour phase delivery of this active ingredient can be recommended as a mitigation strategy against physiological pyrethroid resistance. Nevertheless, it is encouraging that this readily available vapour-phase insecticide also provided significant protection against *Culex spp.* mosquitoes that are known to be highly pyrethroid resistant in Tanzania [60, 61]. Although the level of protection against *Culex spp.* declined steadily with time, it did so quite slowly and estimated mean protection remained satisfactory until the strips were approximately 3 months old (Fig. 2). Although protective efficacy against *An. gambiae s.s*. did not appear to decline with time, this results should be interpreted with a degree of caution, because there was dramatic decrease in the already-low numbers of *An. gambiae* in the untreated group, especially after 10^th^ weeks of sampling. The declining densities of this vector as the treated strips aged undoubtedly reduced the statistical discriminative power of the GLMM fitting process, thus leaving this test for loss of protective efficacy prone to Type II error. Also, the protective efficacy of the strips was not evaluated beyond four months post-treatment, because the primary target vector species for this evaluation (*An. gambiae*) went below detectable levels by the middle of the dry season in August 2012 (Fig. 2). The duration of the protective effect will, therefore, need to be evaluated over longer periods, against vector populations with higher densities that are sustained over correspondingly extended time frames. Long-term evaluation of protective efficacy in a variety of representative field settings will be essential to establish, not only its efficacy and duration of efficacy against a range of relevant vector species, but also overall operational cost (inclusive of retreatment and replacement) per year of protection [62–64] under a wider range of environmental conditions and epidemiological contexts.

It will also be essential to evaluate the dependence of biting exposure decrease or increase upon distance from the point of release of the volatile active ingredients, which is likely to vary depending on delivery format [65]. Also, the chemical concentrations of active ingredient present in the air of protected spaces will need to be measured in future studies, to allow assessment of safety for humans based on existing evidence of toxicity in vapour phase. Potential for environmental contamination of aquatic habitats by such products also merits consideration, especially given recent experience with pyrethroid-treated bed nets [66].

## Conclusions

Despite all these considerable study limitations, it is nevertheless encouraging that this simple, affordable and practical formulation of a volatile pyrethroid that has been available “off-the-shelf” for decades provides such high levels of outdoor protection for such long periods against two of the most important vector species of mosquitoes in the world, without any evidence of diversion to unprotected non-users in nearby houses. Beyond this simple strip prototype, the adaptable nature of such a high capacity absorbent fabric matrix for impregnation with volatile active ingredients also lends itself to a variety of convenient, targeted applications in everyday life, such as curtains or flower pot holders in houses or bars, or layers incorporated into the soles of footwear. However, sociological studies to identify the optimal formats for maximizing community acceptance and coverage will be essential to achieve effectiveness, rather than merely efficacy in practice [67, 68]. So while this study certainly raises more questions than it answers, perhaps the results presented here are encouraging enough to suggest this technology may have potential for preventing outdoor transmission of malaria, LF and other vector-borne pathogens.

## Consent

Written informed consent was obtained from the volunteer for the participation in the study and for the publication of this report and any accompanying images.
